# Drug Resistant *Mycobacterium tuberculosis* of the Beijing Genotype Does Not Spread in Sweden

**DOI:** 10.1371/journal.pone.0010893

**Published:** 2010-05-28

**Authors:** Solomon Ghebremichael, Ramona Groenheit, Alexandra Pennhag, Tuija Koivula, Emmi Andersson, Judith Bruchfeld, Sven Hoffner, Victoria Romanus, Gunilla Källenius

**Affiliations:** 1 Department of Bacteriology, Swedish Institute for Infectious Disease Control, Solna, Sweden; 2 Department of Microbiology, Tumor and Cell Biology, Karolinska Institutet, Stockholm, Sweden; 3 Center for Microbiological Preparedness, Swedish Institute for Infectious Disease Control, Solna, Sweden; 4 Department of Clinical Science and Education, Karolinska Institutet, Södersjukhuset, Stockholm, Sweden; 5 Unit of Clinical Microbiology, (F68), Karolinska University Hospital at Huddinge, Stockholm, Sweden; 6 Infectious Diseases Unit, Department of Medicine, Karolinska Institutet, Karolinska University Hospital, Solna, Sweden; 7 Department of Epidemiology, Swedish Institute for Infectious Disease Control, Solna, Sweden; University of Hyderabad, India

## Abstract

**Background:**

Drug resistant (DR) and multi-drug resistant (MDR) tuberculosis (TB) is increasing worldwide. In some parts of the world 10% or more of new TB cases are MDR. The Beijing genotype is a distinct genetic lineage of *Mycobacterium tuberculosis*, which is distributed worldwide, and has caused large outbreaks of MDR-TB. It has been proposed that certain lineages of *M. tuberculosis*, such as the Beijing lineage, may have specific adaptive advantages. We have investigated the presence and transmission of DR Beijing strains in the Swedish population.

**Methodology/Principal Findings:**

All DR *M. tuberculosis* complex isolates between 1994 and 2008 were studied. Isolates that were of Beijing genotype were investigated for specific resistance mutations and phylogenetic markers. Seventy (13%) of 536 DR strains were of Beijing genotype. The majority of the patients with Beijing strains were foreign born, and their country of origin reflects the countries where the Beijing genotype is most prevalent. Multidrug-resistance was significantly more common in Beijing strains than in non-Beijing strains. There was a correlation between the Beijing genotype and specific resistance mutations in the *katG* gene, the *mabA-inhA*-promotor and the *rpoB* gene. By a combined use of RD deletions, spoligotyping, IS*1547*, *mutT* gene polymorphism and Rv*3135* gene analysis the Beijing strains could be divided into 11 genomic sublineages. Of the patients with Beijing strains 28 (41%) were found in altogether 10 clusters (2–5 per cluster), as defined by RFLP IS*6110*, while 52% of the patients with non-Beijing strains were in clusters. By 24 loci MIRU-VNTR 31 (45%) of the patients with Beijing strains were found in altogether 7 clusters (2–11 per cluster). Contact tracing established possible epidemiological linkage between only two patients with Beijing strains.

**Conclusions/Significance:**

Although extensive outbreaks with non-Beijing TB strains have occurred in Sweden, Beijing strains have not taken hold, in spite of the proximity to high prevalence countries such as Russia and the Baltic countries. The Beijing sublineages so far introduced in Sweden may not be adapted to spread in the Scandinavian population.

## Introduction

Drug resistant (DR) and multi-drug resistant (MDR) tuberculosis (TB) are increasing worldwide. In some parts of the world 10% or more of new TB cases are MDR (i.e. at least resistant to rifampicin (RIF) and isoniazid(INH)) [1], [2]. In the Baltics and Russia a large increase in DR-TB has occurred during the last two decades. Several studies have associated the Beijing genotype of *Mycobacterium tuberculosis* with drug resistance [3]. The Beijing genotype is a distinct genetic lineage [3], which is distributed worldwide, but predominates in certain geographic areas. Phylogenetically the Beijing lineage, defined by spoligotyping, shows the same characteristics as the so called East Asia clade [4], as defined by genomic deletion analysis [5]. The highest prevalence has been detected in parts of Asia [6], [7] and in the former Soviet Union [8], including in prison systems [9].

Both drug susceptible and DR Beijing strains have caused substantial transmission of disease [3]. Beijing strains have caused large outbreaks of MDR-TB [10], [11], and strains of the W Beijing genotype caused a large outbreak of MDR-TB in the United States in the early 1990s [10]. Further on members of the same family of strains were observed in many other institutional and nosocomial outbreaks and in ongoing community transmission [7].

The ubiquity of Beijing strains and an association with multidrug-resistance [12] has lead to concern that they possess a unique ability to acquire drug resistance. Such associations have been found in e.g. Vietnam, Germany, Cuba, the US, Russia and Estonia [1], [3]. However, the endemic Beijing strains in China and Mongolia are pan-susceptible [3]. In Estonia genetically closely related Beijing strains showed a range from full susceptibility to four-drug resistance, indicating that drug resistance had developed recently and independently in different clones of Beijing strains [13].

Sweden has 9 million inhabitants, of which about 10% are foreign born. The TB incidence is low, with an incidence of 6.0 per 100,000 population in 2008, and more than two thirds of new TB cases being foreign born. Drug resistance has increased during the last five years. In 2008 drug resistance was observed in 13.1% of all culture confirmed cases, and multidrug-resistance in 3.2%.

We have previously reported the spread of resistant TB in Sweden [14]. Of 400 isolates, collected during 1994–2005, 48 (12%) isolates were of the Beijing family. We here describe the Beijing strains in more detail, extending the study to 2008. Our aim was to investigate the prevalence and possible transmission of Beijing TB strains in Sweden in relation to drug resistance.

## Methods

### Ethics Statement

At the Swedish Institute for Infectious Disease Control (SMI), *M. tuberculosis* strains are routinely collected for disease surveillance. The current study describes a bacterial collection and bacterial genotypes could only be combined with the sex, age, and country of birth for the patients from which the strains were isolated. Ethical approval was therefore not required. For the same reason, consent was not obtained from the patients to analyze the bacterial samples for this population based retrospective study.

### Patients and isolates

During the years 1994–2008, all patients in Sweden with drug resistant TB were reported to SMI. An investigation of contacts was routinely conducted by the attending physician and reported to the County Departments of Communicable Disease Control and Prevention. Patients infected with organisms with identical RFLP patterns were studied further by a more intensive review and contact tracing to identify possible epi-links. For this study, information on epi-links obtained by conventional contact tracing was retrospectively collected by the Department of Epidemiology at SMI.

DR *M. tuberculosis* isolates were obtained from all Swedish TB laboratories, situated in Gothenburg, Linköping, Malmö/Lund, Stockholm and Umeå. The isolates and patients from 1994–2005 have been previously described [14]. Isolates resistant to at least one of the drugs, INH, RIF, ethambutol (EB) or streptomycin (SM) were included. In Sweden, all isolates are tested for susceptibility to the first-line drugs INH, EB and RIF. During the major part of the study all isolates were also tested for susceptibility to SM, except for the years 2004–2008, when two laboratories stopped routine testing for SM-resistance. The isolates were typed and drug susceptibility testing was performed with standard methods. All laboratories had taken part in the external quality assurance program for drug susceptibility testing of *M. tuberculosis* offered by the Swedish TB reference laboratory at SMI. The first isolate from each patient identified as resistant to one or more of these drugs was included in the study. In one case two different isolates of different genotype from one patient were included.

### Spoligotyping

All isolates were characterized by spoligotyping, which characterizes the polymorphic direct repeat region of the *M. tuberculosis* chromosome [15]. The patterns obtained by spoligotyping were compared by visual examination and by sorting the results in BioNumerics software version 5.10 (Applied Maths, Kortrijk, Belgium). The spoligotypes were also compared with those contained in the international database SITVIT2, an updated variant of the previously published SpolDB4 database [16], and were assigned to the major phylogenetic lineages according to signatures provided in SITVIT2, which defines 62 genetic lineages/sublineages of *M. tuberculosis* complex isolates (http://www.pasteur-guadeloupe.fr:8081/SITVITDemo). The SITVIT2 database contains to date more than 73.000 spoligopatterns for clinical isolates, with more than 3.000 spoligotype international types (SITs; a pattern shared by two or more patient isolates). By spoligotyping isolates of the Beijing genotype were defined by showing hybridization to at least three of the spacers between spacers 35 to 43 and showing the absence of hybridization to spacers 1 to 34 [17]. Some Beijing strains lack one or more of the nine signature spacers, due to asymmetrical insertions of IS*6110* [18].

### IS*6110*-RFLP

The isolates were cultured on Löwenstein-Jensen medium, DNA was extracted and RFLP typing was performed using the insertion sequence IS*6110* as a probe and *Pvu*II as the restriction enzyme [7], [19]. Visual bands were analyzed using the BioNumerics software version 5.10 (Applied Maths, Kortrijk, Belgium). Strains with identical RFLP patterns (100% similarity) and five or more hybridizing bands, were judged to belong to a cluster. On the basis of the molecular sizes of the hybridizing fragments and the number of IS*6110* copies of each isolate, fingerprint patterns were compared by the un-weighted pair-group method of arithmetic averaging using the Jaccard coefficient. Dendrograms were constructed to show the degree of relatedness among strains according to a previously described algorithm [20] and similarity matrixes were generated to visualize the relatedness between the banding patterns of all isolates.

### IS*1547*-RFLP

Southern hybridization was done by using the same blots (*Pvu*II - digested DNA) as for IS*6110*-RFLP. The IS*1547* probe for hybridization was obtained by amplification using the primers 15F (5′-TGTGTGTGCCGCGAGGTGGG-3′), and 15R (5′-GCAATAGCTCCTATGGCAAGCGGC-3′) [21], [22].

### Mycobacterial Interspersed Repetitive-Unit-Variable-Number Tandem-Repeat (MIRU-VNTR) Genotyping

Standardized 24-locus MIRU-VNTR typing [23] was performed using the MIRU-VNTR typing kit (Genoscreen, Lille, France). The PCR-products were run with 1200 LIZ size standard (GeneScan, Applied Biosystems) on ABI3131xl sequencers. Sizing of the PCR-fragments and assignments of MIRU-VNTR alleles were done with the GeneMapper software version 4.0 (Applied Biosystems) according to the manufacturers' instructions.

### Genotyping for specific resistance mutations

#### Analysis of the *katG* gene

DNA sequencing of the 691-bp fragment of *katG* was performed using primers F768 *katG* (3′-CATGAACGACGTCGAAACAG-5′) and R1458 *katG* (3′-GCTACCACGGAACGACGAC-5′).

#### Analysis of the *mabA*-*inhA*-promotor

DNA sequencing of the 980-bp fragment of the *inhA* promotor region was performed using primers *inhA*-F (5′-TCGTAGGGCGTCAATACACCGCA-3′) and *inhA*-R (5′-CGTCCAGCAGTCCTGTCATGTGCGT-3′) as previously described [24].

#### Analysis of the *rpoB* gene

In RIF resistant strains a 382-bp fragment of the *rpoB*-gene containing the RIF resistance-determining region (RRDR) [25], [26] was sequenced using primers OPRIF-F (5′-CGGTCGGCGAGCTGATCC-3′) and OPRIF-R (5′-TGGACCCGCGCGTACACC-3′) as previously described [27]. Cycle sequencing was performed using The BigDye Terminator v3.1 Cycle Sequencing Kit (Applied Biosystems, Sweden). Sequences were compared to those of the wild-type genes of strain H37Rv (http://www.ncbi.nlm.nih.gov) using the BioEdit Sequence Alignment Editor.

### RD polymorphism

Genomic deletions RD105, RD142, RD150 and RD181 were identified by PCR using primer sets as previously described [5] and the PCR products were analysed by agarose gel electrophoresis.

### Rv*3135* gene analysis

Rv*3135* gene analysis was performed as previously described [22], [28] using primers: Rv*3135*-F (5'-TCGACTGCCATACAACCTG-3') and Rv*3135*-R (5'-GTGCTGGTCGAGAACTGAATG-3') located 210 bp upstream from the Rv*3135* start site and 23 bp downstream from the stop codon, respectively. These primers amplify a 632-bp product from strain H37Rv.

### *mutT* gene polymorphism

Mutation analysis of *mutT2* and *mutT4* was performed by nucleotide sequencing of these genes. Amplification was carried out using primers [29] generating fragments of ∼800 and ∼1000 bp that were visualized on a 1% agarose gel. PCR products were purified using spin columns (GE Healthcare). The mutations GGA to CGA (Gly to Arg) at codon position 58 for *mutT2* and CGG to GGG (Arg to Gly) at codon position 48 for *mutT4* was detected using the ABI Big Dye v. 3.1 cycle sequencing kit.

### Subgrouping of Beijing strains – genomic sublineages

Based on the results of RD deletion analysis, spoligotyping, IS*1547, mutT* gene polymorphism and Rv*3135* gene analysis the 70 Beijing strains were allocated to genomic sublineages, where each sublineage represented a unique genetic setup according to these analyses.

### Review of patient data

Available demographic data were collected for all patients infected with Beijing strains, such as age, gender and country of origin.

Statistical associations were generated by MINITAB Version 15 using χ^2^ (Chi-square) test. For analysis of epidemiological trends over time the Sas ARIMA Model was used.

## Results

### Patients

Altogether 536 DR isolates from 535 patients were collected in 1994–2008. Sixty-nine (13%) of the 535 patients with DR-TB were found to have strains of Beijing genotype as defined by spoligotyping.

In one case multiple strains were found in the same patient. The patient, born in 1970, had in May 2003 a sputum sample from which three different isolates were cultured, one of which was of Beijing genotype and resistant to INH. In December 2003 the same patient presented with a sample containing yet another Beijing strain with a different RFLP pattern. The second isolate was resistant to all four drugs. This patient came from Azerbaijan, and was reported to have been in prison.

**Figure 1:**
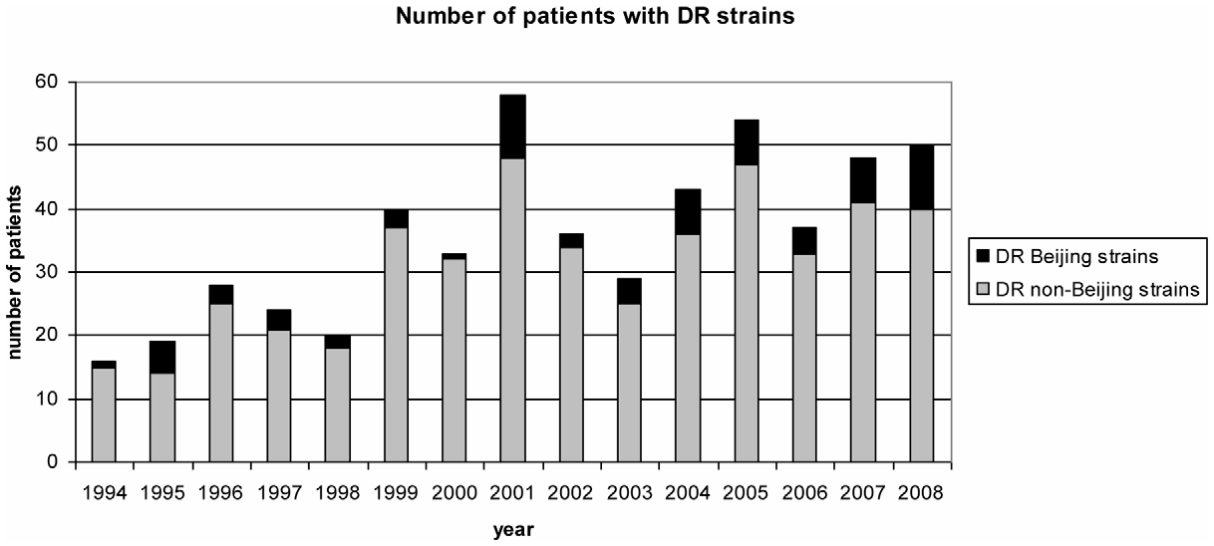
Number of patients with DR strains (Beijing and non Beijing) isolated over time. A trend of increase over the years (p = 0.0175, significance level of 5%) was observed for DR Beijing isolates as well as for all DR isolates (p = 0.0421, significance level of 5%), using the Sas ARIMA Model for analysis of epidemiological trends over time.

A median of four patients with DR Beijing strains per year was observed, with a slight trend of increase over the years (p = 0.0175, significance level of 5%) following the overall increase in DR isolates (p = 0.0421, significance level of 5%) during those years (Fig. 1). Of the 69 patients with DR Beijing strains 6 (8.7%) were born in Sweden and 63 born abroad. Of the 466 patients with DR non-Beijing strains 63 (13.5%) were born in Sweden and 403 born abroad. Forty-two (67%) of the 63 foreign born TB-patients infected with DR Beijing strains were women and 27 were men (Table 1). The majority of the patients with DR Beijing strains (58%) were in the age group of 25–44 and 20% in the age group 15–24 (Table 1), which is consistent with the age distribution for patients with DR-TB in general (Table 1) in Sweden. Patients with Beijing strains were more often women than were those infected with strains of other genotypes (p-value = 0.0268, using a one-sided test of hypothesis at 5% significance level).

**Table 1:** Age and sex of 535 patients with drug resistant strains, and relative proportion of patients with Beijing strains.

	Male			Female		
Age (years)	total	Beijing	%	total	Beijing	%
0–14	14	0	0	16	1	6.3
15–24	50	4	8	63	10	15.9
25–44	143	16	11.2	136	24	17.6
45–64	48	7	14.6	32	4	12.5
65+	12	0	0	21	3	14.3
Total*	267	27	10.1	268	42	15.7

*Patients with Beijing strains were more often women than were those infected with strains of other genotypes (p-value = 0.0268, using a one-sided test of hypothesis at 5% significance level).

### Country of origin

Of the 69 patients with DR Beijing strains that were foreign born a large proportion came from Asia (75%); the majority of these patients were from Vietnam (n = 19) and Thailand (n = 7), which are the two single countries that contributed with most patients with Beijing strains (Supporting material Table S1). Most of the patients from Asia with DR-TB were found to have strains of Beijing genotype. Thus, 59% of patients from Vietnam, 54% of patients from Thailand and all (altogether n = 17) patients with DR-TB from Armenia, Azerbaijan, Bhutan, Cambodia, China, Kazakhstan, Korea, North Korea and Uzbekistan had strains of Beijing genotype. Only 11 (4%) of 280 patients from Africa with DR-TB had Beijing strains.

Of the six Swedish born patients that were infected with DR Beijing strains three had parents who were born outside Sweden (Tibet, Hungary and Chile). The Swedish born patient with parents with origin in Tibet, probably was infected while visiting Tibet.

### Strains

Of the 70 strains that were defined by spoligotyping to be of the Beijing genotype 66 had all the characteristic spacers 35–43, corresponding to the shared type SIT1 as defined in SITVIT2, and four isolates lacked spacer 37, corresponding to SIT265 (Table 2). By RFLP the isolates had 13 to 23 IS*6110* insertions.

**Table 2:** Polymorphims of *M. tuberculosis* strains of Beijing genotype.

			Region of difference (RD)^a^					Mutation in m*utT* genes^b^		
Genomic sublineage	Number of isolates (n = 70)	SIT^c^	105	181	150	142	Rv*3135* d	*mutT2*	*mutT4*	IS*1547* e
1	1	265	+	+	+	+	0.15 kb	−	−	8
2	13	1	−	−	+	+	at	−	+	5
3	1	1	−	−	+	+	at	−	+	6
4	1	1	−	−	+	+	at	−	+	7
5	41	1	−	−	+	+	t	+	+	1
6	3	1	−	−	+	+	t	+	+	2
7	1	1	−	−	+	+	t	+	+	3
8	3	265	−	−	+	+	t	+	+	1
9	1	1	**−**	−	+	−	t	+	+	1
10	4	1	−	−	−	+	t	+	+	1
11	1	1	−	−	−	+	t	+	+	4

Isolates are grouped into 11 arbitrary sublineages on the basis of the polymorphisms relative to genomic deletion RD105, RD181, RD150 and RD142, RV3135 IS*1547* RFLP pattern and mutations in genes *mutT4* and *mutT2*.

a presence (+) or absence (−) of the specific genomic region.

b presence (+) or absence (−) of the specific mutation.

c spoligotype international type.

d sublineage 1: a 0.15-kb PCR product, sublineage 2–4: “atypical” isolates, with a 1.97-kb PCR product, sublineage 5–11: “typical” isolates, with a 1.02-kb PCR product.

e pattern 1: three bands (1.7, 2.1, and 2.5 kb), pattern 2: two bands (2.1 and 2.5 kb), pattern 3–7: individual patterns, all containing the 2.5 kb band, except pattern 8 that has no 2.5 kb band.

### Drug resistance

Of the 70 Beijing strains 51 were INH resistant, 18 were resistant to RIF, and 17 were MDR. One strain was defined as extensively drug resistant (XDR), in this case resistant to amikacin and ofloxacin. Thirty-three strains were monoresistant, either to SM (n = 17), to INH (n = 14), to RIF (n = 1) or to EB (n = 1). While 17 (24%) of the Beijing strains were MDR, only 59 (13%) of 466 DR non-Beijing strains were MDR during the same period, which is a statistically significant difference [p-value 0.0134, 95% CI = (0.0137; 0.2258)].

### Genotyping for specific resistance mutations

Resistance mutations in the *katG* gene and/or in the *inhA* promoter region were found in 49/51 (96%) of the Beijing strains with phenotypic INH resistance (Table 3). Forty-four isolates (86%) had a mutation in *katG*, showing a nucleotide exchange in *katG* codon 315 from AGC to ACC. Eight isolates (16%) had a mutation in the *inhA* promoter region showing an exchange from C to T at position -15. Three of the strains (including the XDR strain) had both mutations. All 17 MDR strains had the 315ACC mutation in the *katG* gene. Resistance mutations in the RIF resistance-determining region of the *rpoB* gene were found in 16 of 18 strains with phenotypic RIF resistance. Mutation in *rpoB* codon 531 was most common (n = 13).

**Table 3:** Resistance related mutations in Beijing strains.

MDR isolates n = 17	*katG*	*inhA*	*rpoB*
n = 10	Ser315Thr	wild-type	Ser531Leu
n = 2	Ser315Thr	wild-type	wild-type
n = 2	Ser315Thr	C(-15)T	Ser531Leu
n = 1	Ser315Thr Ile335Val	wild-type	Ser531Leu
n = 1	Ser315Thr	wild-type	Asp516Val
n = 1	Ser315Thr	wild-type	Asp516Tyr

INH monoresistant isolates n = 34	*katG*	*inhA*	*rpoB*
n = 26	Ser315Thr	wild-type	ND
n = 5	wild-type	C(-15)T	ND
n = 2	wild-type	wild-type	ND
n = 1	Ser315Thr	C(-15)T	ND

RIF monoresistant isolates n = 1	*katG*	*inhA*	*rpoB*
n = 1	wild-type	wild-type	Leu533Pro

All strains (n = 52) harbored the mutation Arg463Leu. ND: not done.

### RD deletions of Beijing strains

The isolates were analysed for the four deletions RD105, RD181, RD150, and RD142 [5]. The majority (n = 69) of the strains had the RD105 and RD181 deletions. One strain had in addition to those two deletions also the RD142 deletion, and five strains had the two deletions plus the RD150 deletion. One strain (BTB04-003) had all RD intact, including RD105.

### IS*1547* polymorphism

Eight different patterns were seen. All strains except one (BTB04-003) had a band of 2.5 kb. This band has been reported to be common to all Beijing strains [22]. A single IS*1547*-RFLP band (2.5 kb) pattern was observed for 13 strains, corresponding to the pattern of “atypical” strains described by Mokrousov et al [22] while a three band (1.7, 2.1, and 2.5 kb) pattern was found for 49 strains, corresponding to the pattern of “typical” Beijing strains [22]. Three strains had a two band (2.1 and 2.5 kb) pattern, while five strains had individual patterns, all containing the 2.5 kb band.

### Rv*3135* gene analysis

PCR of the Rv*3135* genome region showed a 1.02-kb fragment in 54 strains, and a 1.97-kb fragment in 15 strains. One strain (BTB04-003) had a PCR fragment of around 0.15-kb.

### *mutT* gene polymorphism

All except one strain (n = 69) had the *mutT4* mutation, and 53 isolates had the *mutT2* mutation.

### Genomic sublineages

By a combined use of RD deletions, spoligotyping, IS*1547*, *mutT* gene polymorphism and Rv*3135* gene analysis the 70 Beijing strains could be divided into 11 arbitrarily numbered genomic sublineages (Table 2). Fifty-four strains (7 sublineages) fulfilled the criteria of being “modern”, or “typical” Beijing isolates according to Rv*3135* gene analysis [22]. Of the 16 more “ancient” or “atypical” Beijing strains 7 strains clustered in 3 different IS*6110* RFLP clusters, while 21 of the 54 “modern” strains clustered in 7 different RFLP clusters.

One strain (BTB04-003) lacked all the properties of a modern Beijing strain, except for its SIT265 spoligotype. It had all RD intact, had wildtype *mutT2* and *mutT* genes, and did not have the IS*1547* 2.5 kb band, which is regarded as specific for Beijing strains [22]. It even lacked the RD105 deletion which is considered to be a marker for Beijing strains.

### Epidemiological links

**Figure 2:**
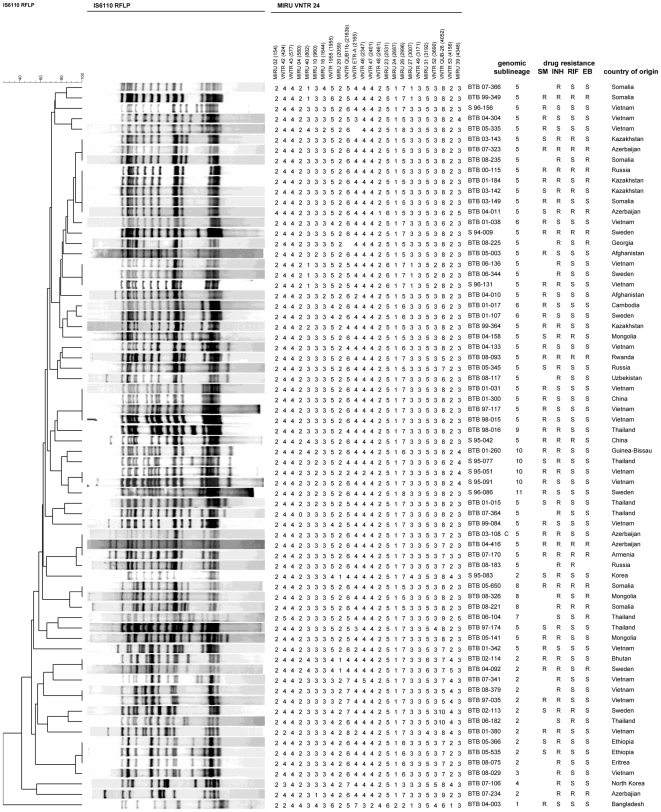
IS*6110* dendrogram of 70 *M. tuberculosis* Beijing strains. The IS*6110* RFLP dendrogram was constructed to show the degree of relatedness among strains according to [20] and similarity matrixes were generated to visualize the relatedness between the banding patterns of all isolates. MIRU-VNTR  =  Mycobacterial Interspersed Repetitive-Unit-Variable-Number Tandem-Repeat. The genomic sublineages were arbitrarily defined on the basis of the combined use of RD deletions, spoligotyping, IS*1547, mutT* gene polymorphism and Rv*3135* gene analysis (Table 2). SM = streptomycin, INH = isoniazid, RIF = rifampicin, EB = ethambutol, R = resistant, S = susceptible.

In this study 28 (41%) of the 69 patients with Beijing strains were found in altogether 10 clusters (2–5 per cluster) as defined by IS*6110* RFLP, yielding 51 different patterns (Fig. 2) while 240 patients (52%) of 466 patients with non-Beijing DR-TB strains during the same time period were in clusters. In three clusters the patients with Beijing strains were from the same country of origin: two, two and four patients respectively from Vietnam. The largest cluster (SMI 009) comprised five patients. Four of the patients in this cluster were from the former Soviet Union, three of them having MDR strains. The fifth patient in this cluster came from Somalia. All strains that clustered by IS*6110*-RFLP were identical also by RD deletions, spoligotyping, IS*1547*, *mutT* gene polymorphism and Rv*3135* gene analysis. Of the six Swedish born patients two were in clusters, one with two patients from Vietnam, and one with a patient from Bhutan. Contact tracing established possible epidemiological linkage for one of the clusters (cluster SMI 176). This was the cluster comprising the two patients from Sweden and Bhutan. The two men had shared the same indoor recreational activity during the wintertime of 2002. In the other clusters no epidemiological link was found by conventional contact tracing.

By MIRU-VNTR 31 (45%) of the 69 patients with Beijing strains were found in altogether 7 clusters (2–11 per cluster), yielding 45 different patterns (Fig. 2). Thus the MIRU-VNTR typing, with fewer and larger clusters, was less discriminatory than IS*6110* RFLP. The two strains where a possible epidemiological linkage was established differed in one allele and thus did not cluster in MIRU-VNTR. All strains that clustered by MIRU-VNTR were identical also by RD deletions, *mutT* gene polymorphism and Rv*3135* gene analysis, but not by spoligotyping and IS*1547*.

Four of the IS*6110* RFLP clusters contained isolates that differed by MIRU-VNTR (Fig. 2). The combination of MIRU-VNTR with RFLP resulted in the disappearance of two clusters, and a reduction of the number of isolates in two clusters, compared to the clustering observed with IS*6110* RFLP clustering alone.

## Discussion

In this study we found that patients with DR Beijing strains have been diagnosed for more than a decade in Sweden. The majority of the patients were foreign born, and their country of origin reflects areas where the Beijing genotype is prevalent.

The Beijing genotype has been reported to be most prevalent in Asia [3], [7], [30] and parts of the former Soviet Union [3]. In this study patients with DR Beijing strains were mainly from Asia (75%); the majority of patients from Asia being from Vietnam and Thailand. This is in accordance with the reports of Beijing strains predominating in China and neighbouring Asian countries such as Thailand [7] and Vietnam [31]. In Vietnam the proportion of Beijing strains was reported to be 54% [31], and in Thailand 44% [32].

There were only a few patients with Beijing strains from the former Soviet Union, reflecting the fact that only a few TB patients originating from the former Soviet Union were reported in Sweden during this period [33]. Yet, all DR isolates from patients from Kazakhstan and Azerbaijan were of Beijing genotype, which conforms to the high prevalence of the Beijing genotype reported in the former Soviet Union [1], [13], [34], [35].

Beijing strains are however still uncommon in Africa, except for South Africa [36]. Only 11 (16%) of the patients with DR Beijing strains were from Africa, although as much as 52% of 535 patients with DR-TB in Sweden were of African origin. Thus, the prevalence of the Beijing genotype in Sweden appears to reflect the prevalence of the Beijing genotype in the country of origin of the patients as well as the proportion of TB patients from these countries.

### Age and sex

Patients with Beijing strains were significantly more often women than were those infected with strains of other genotypes. In only one study such gender difference has been previously recorded [37]. The higher number of female patients with Beijing strains might reflect the larger immigration of women than of men from some countries with a high prevalence of Beijing strains. Thus there were more women than men from Thailand and Vietnam, also among those with non-Beijing strains.

### Simultaneous infections

One patient harboured at least four different strains, of which two were of Beijing genotype. The patient came from a high endemic area where Beijing strains are circulating. This confirms previous findings reporting mixed infections [35], [38], [39], [40], [41], [42], [43], [44]. Interestingly, most mixed infections reported involved Beijing strains, sometimes in combination with non-Beijing strains, and the patients came from areas where they had been heavily exposed to TB.

### Strains of Beijing genotype more often MDR

In this study the prevalence of multidrug-resistance was significantly higher in Beijing strains than in DR non-Beijing strains, which is in accordance with multiple reports of an association between the Beijing genotype and drug resistance and multi-drug resistance [13], [45], [46] Thus, in Central Asia and the former Soviet Union the Beijing genotype is associated with drug and multidrug-resistance [12], [45], which is reflected in a high prevalence of DR Beijing strains in patients from these countries. In this study there was only one isolate with mono-resistance to RIF, which is in agreement with previously published data [47], indicating that the ability to acquire INH resistance is the first and most important step to acquire MDR TB in most instances [48].

### Drug resistance genotypes

Several studies indicate an association between Beijing genotype and mutations in the *katG* gene [3], [49], [50]. The majority of the 51 strains with INH resistance had the *katG* 315 substitution, while five isolates had the -15 *inhA* mutation in the promoter of the *inhA* gene, which encodes an enoyl acyl carrier protein reductase involved in fatty acid synthesis [51]. In Northwestern Russia all Beijing isolates from new cases that were INH resistant had the *katG* 315 substitution [49]. In a study from Kazakhstan [43] all MDR strains, irrespective of genotype, carried the mutation at codon 315 of the *katG* gene, and in a group of INH resistant but RIF sensitive strains, this mutation was significantly more prevalent in the Beijing than in the non-Beijing group of strains [43]. The *katG* 315 mutation was more common among Beijing vs. non-Beijing strains in a study from Russia (96.8% vs. 85.7%) [52] and Korea (65% vs. 50%)[46], while the -15 *inhA* mutation was less common among Beijing strains (14% vs. 25% for non-Beijing strains) [46].

However, in a study of children with DR TB in South Africa, the -15 *inhA* promoter region mutation was the most frequent mutation (59%) in INH resistant Beijing strains [47]. One reason for the predominance of this mutation in the study from South Africa could be that it was exclusively detected in the Beijing 220 subgroup, a well-recognized DR strain in the Western Cape Province [47], indicating a clonal dissemination of this DR strain. Interestingly, in our study four of the five Beijing strains with the -15 *inhA* promoter region mutation occurred in patients from Africa, and seven of the eleven patients from Africa (Somalia, Ethiopia and Rwanda) with INH resistant strains had the *katG* 315 substitution.

Most of the RIF resistant Beijing strains had a mutation at the 531 codon of *rpoB*. This is in accordance with a previously reported strong association between Beijing strains with multidrug-resistance and mutations at codon 531 [12], [50], [53]. Thus the *rpoB* 531 mutation was more prevalent in MDR Beijing than non-Beijing strains from Russia [52] (77.3% vs. 28%) and Kazakhstan (71.2 vs. 46.2%) [43]. On the other hand a study from Korea showed lower rates of the *rpoB* 531 mutation in Beijing vs. non-Beijing MDR-TB strains (41 vs. 66%) [46].

Interestingly the only RIF mono-resistant strain (isolate BTB06-182, sublineage 2) from a patient originating from Thailand carried a rare mutation at the 533 codon of the *rpoB* gene. Resistance to RIF in most cases occurs in combination with INH resistance, and in the few available reports of RIF mono-resistance the mutations underlying the resistance are rarely observed in MDR strains. An unusual duplication at codon 415 was observed in a mono-resistant strain spreading in an HIV-seropositive population [54]. In a study from Thailand, the prevalence of the common mutations in codon 531 was lower in RIF monoresistant strains than in MDR strains [55].

### Association between genotype and specific resistance mutations

From all these studies, there appears to be a correlation between the Beijing genotype and specific resistance mutations. Few studies have reported on correlations between other genotypes and mutations in resistance genes [56]. In a study from Russia, there appeared to be a correlation between the genotype and specific mutations conferring resistance to RIF [57]. Another study of MDR *M. tuberculosis* isolates from India [53]only showed an association between *rpoB* 531 mutations and Beijing genotype, while the type of mutations in the *rpoB* gene were independent of genotype (spoligopattern) in non-Beijing strains.

### Genomic lineages and adaptive advantage

This collection of Beijing strains from various parts of the world illustrates the genomic diversity of the Beijing family. Based on a number of genetic markers the 70 Beijing isolates could be allocated into 11 different arbitrary sublineages, from more “modern”, or “typical” [22] to more “ancient” or “atypical” [22] variants.

The particular nature of strain BTB04-003, isolated from a man from Bangladesh, which lacked all the properties of a modern Beijing strain, except for its SIT265 spoligotype, remains to be elucidated. The lack of deletion of RD105, which is considered to be a marker for Beijing strains [5], [58], has been reported in one other strain [59]. This strain was assigned to the spoligotype SIT269, which is defined as “Beijing-like” in SITVIT2. The deletion of the RD105 region was recently found also in ancestral strains with non-Beijing spoligoprofiles [60]. The BTB04-003 strain may be either a very early ancestral variant, occurring before the RD105 deletion, or may have developed independently through selective pressure.

### Epidemiological links

Contact tracing revealed a possible connection between only two of the patients in the ten IS*6110* RFLP clusters. In this cluster the strains were not identical by MIRU-VNTR. The discordance observed between IS*6110* RFLP and MIRU-VNTR is in agreement with a study of Beijing strains in South Africa [61], where molecular epidemiological linking of cases varied significantly depending on the genotyping method used. Isolates with the same fingerprinting pattern are usually thought to reflect ongoing transmission. However, Beijing strains exhibit restricted variation in RFLP patterns. It was recently shown [62] that two Beijing strains exhibiting the same fingerprinting pattern harbored substantial genomic diversity. The fact that strains with identical genotyping patterns can accumulate significant amounts of genetic diversity indicates that epidemiological links between strains with identical genotyping data can be more remote and are likely to represent older transmission events rather than cases of recent transmission among patients in one RFLP cluster[62]. In this study four of the 10 RFLP clusters could be further resolved on the basis of MIRU-VNTR data, resulting in the disappearance of two clusters, and the reduction of the number of strains in two clusters. Two more clusters had strains with different drug resistance genotypes. Thus the clustering by conventional IS*6110* RFLP in this study in most cases probably reflects the fact that the patients in the individual cluster came from the same geographical area, and had strains of genotypes that were prevalent in their respective countries of origin.

### Little spread of the Beijing family in Scandinavian countries

The global distribution of Beijing strains and their ability to predominate suggests that they have advantages to other strains that make them better adapted to infect and cause disease in humans. It has been proposed that certain lineages of *M. tuberculosis*, such as the Beijing lineage, may have specific adaptive advantages [4], [63], [64]. Active transmission of Beijing strains has been described in the US [65], Estonia [13] and the Canary Islands [66]. There is a recent and rapid emergence of Beijing strains in Cape Town, SA [67], and a recently evolved sublineage of the Beijing family has been associated with an increased ability to spread and cause disease in South Africa [58]. Yet, in spite of the closeness to Russia and the Baltic states Beijing strains have not been observed to spread extensively within any of the Scandinavian countries. In Finland, that is most closely geographically situated to both Russia and Estonia, only 1% of patients were infected with a Beijing strain in 2001 [68]. In Denmark 1% of Danish born and 3.6% of immigrant TB patients had Beijing strains [69]. No evidence of an increase in Beijing strains was found over time, and very little transmission from immigrants to the indigenous populations was observed. In this population based study, in spite of a steady influx of Beijing strains, no, or very little spread of Beijing strains within Sweden could be documented.

The incidence of Beijing strains is low in most Western European countries, although strains of the Beijing genotype represent an increasing cause of MDR-TB in Germany [41]. The low incidence of Beijing strains in the Scandinavian countries may indicate that there are efficient national TB control programs. On the other hand, large outbreaks with other, non-Beijing, strains have been reported among immigrants in Sweden [13], Norway [70] and Denmark [71]. Thus an INH resistant non-Beijing *M. tuberculosis* strain caused an outbreak in Sweden, now involving more than 100 patients, mainly immigrants from the Horn of Africa [33], and another INH resistant clone caused an outbreak in East African patients in Norway [70]. In both cases eventually MDR variants emerged, also in the native population [14], [70].

It has been suggested that *M. tuberculosis* complex lineages are adapted to particular human populations [4], [64], [72], [73], and that lineages that are rare in a specific human population are not adapted to transmit and cause secondary cases in this specific population [4]. In San Francisco, patients infected with isolates of the Euro-American lineage (as defined by lineage specific DNA polymorphisms [4]) were three times more likely to generate a secondary case than patients infected with any other strain, while the East-Asian (Beijing) lineage had the lowest secondary case-rate ratio [4]. It is assumed that the modern Beijing strains have a higher degree of transmissibility than older, atypical variants. The spread of a modern Beijing sublineage that has a high transmissibility is currently increasing in Japan, while the spread of an ancient sublineage has decreased [74]. In South Africa a recently evolved sublineage of the Beijing strain family was associated with an increased ability to spread and cause disease[58]. It is possible that the Beijing sublineages introduced in Sweden so far are not adapted to spread in the Scandinavian or immigrant populations.

## Supporting Information

Table S1 Geographical origin and sex of 535 patients with drug resistant *M. tuberculosis* isolates.
(0.10 MB DOC)
