# Supplementary material for: Drug Resistant Mycobacterium tuberculosis of the Beijing Genotype Does Not Spread in Sweden
Source: PLoS One. 2010 May 28;5(5):e10893. doi: 10.1371/journal.pone.0010893 (PMC2878347; doi:10.1371/journal.pone.0010893)
Supplement: Table S1 — Geographical origin and sex of 535 patients with drug resistant M. tuberculosis isolates. (0.10 MB DOC) [file pone.0010893.s001.doc]

## Table S1. Geographical origin and sex of 535 patients with drug resistant *M. tuberculosis* isolates

|  | **Males** | | **Females** | |
| --- | --- | --- | --- | --- |
| **Origin** | **Total** | **Beijing** | **Total** | **Beijing** |
| Afghanistan | 4 | 1 | 4 | 1 |
| Armenia | 1 | 1 |  |  |
| Azerbajian | 3 | 3 | 1 | 1 |
| Bangladesh | 2 |  | 2 | 1 |
| Belarus |  |  | 1 |  |
| Bhutan | 1 | 1 |  |  |
| Bosnia | 3 |  | 2 |  |
| Brazil |  |  | 2 |  |
| Burundi | 2 |  | 1 |  |
| Cambodia |  |  | 1 | 1 |
| Chile | 1 |  |  |  |
| China |  |  | 2 | 2 |
| Congo |  |  | 2 |  |
| Cuba |  |  | 1 |  |
| Denmark | 1 |  |  |  |
| Ecuador | 1 |  |  |  |
| El Salvador | 1 |  |  |  |
| Eritrea | 13 |  | 13 | 1 |
| Ethiopia | 14 |  | 13 | 2 |
| Finland | 2 |  |  |  |
| Gambia | 4 |  |  |  |
| Georgia | 1 | 1 | 1 |  |
| Ghana | 2 |  |  |  |
| Guinea-Bissau |  |  | 1 | 1 |
| India | 4 |  | 4 |  |
| Iran |  |  | 11 |  |
| Iraq | 7 |  |  |  |
| Ivory Coast | 1 |  |  |  |
| Kazakstan | 1 | 1 | 4 | 4 |
| Kenya | 4 |  | 2 |  |
| Korea |  |  | 1 | 1 |
| Kosovo | 4 |  | 3 |  |
| Lebanon | 2 |  | 1 |  |
| Malaysia |  |  | 1 |  |
| Mongolia | 1 | 1 | 3 | 2 |
| Morocco |  |  | 1 |  |
| Nigeria | 1 |  |  |  |
| North Korea |  |  | 1 | 1 |
| Pakistan | 2 |  | 1 |  |
| Peru | 6 |  | 2 |  |
| Philippines | 2 |  | 11 |  |
| Poland | 2 |  | 1 |  |
| Rumania | 3 |  |  |  |
| Russia | 3 |  | 3 | 2 |
| Rwanda |  |  | 2 | 1 |
| Somalia | 98 | 3 | 94 | 3 |
| South Africa | 1 |  |  |  |
| Sudan | 1 |  | 1 |  |
| Sweden | 35 | 4 | 34 | 2 |
| Syria | 2 |  | 1 |  |
| Thailand | 3 | 2 | 10 | 5 |
| Tjetjenia | 1 |  |  |  |
| Tunisia | 1 |  |  |  |
| Turkey | 1 |  | 4 |  |
| Uganda | 4 |  | 1 |  |
| Uzbekistan | 1 | 1 |  |  |
| Vietnam | 14 | 8 | 18 | 11 |
| Yugoslavia | 2 |  | 4 |  |
| Zaire | 2 |  | 1 |  |
| Unknown | 2 |  | 1 |  |
| **total** | **267** | **27** | **268** | **42** |
